# The Effect of Online Effort and Reputation of Physicians on Patients’ Choice: 3-Wave Data Analysis of China’s Good Doctor Website

**DOI:** 10.2196/10170

**Published:** 2019-03-08

**Authors:** Zhaohua Deng, Ziying Hong, Wei Zhang, Richard Evans, Yanyan Chen

**Affiliations:** 1 Smart Health Institute School of Medicine and Health Management Huazhong University of Science and Technology Wuhan China; 2 College of Engineering, Design and Physical Sciences Brunel University London United Kingdom; 3 Tongji Hospital, Tongji Medical College, Huazhong University of Science and Technology Wuhan China

**Keywords:** physician-rating websites, physician efforts, physician reputations, patient choices, panel data analysis

## Abstract

**Background:**

Nowadays, patients are seeking physician information more frequently via the internet. Physician-rating websites (PRWs) have been recognized as the most convenient way to gain insight and detailed information about specific physicians before receiving consultation. However, little is known about how the information provided on PRWs may affect patients’ decisions to seek medical advice.

**Objective:**

This study aimed to examine whether the physicians’ online efforts and their reputation have a relationship with patients’ choice of physician on PRWs.

**Methods:**

A model, based on social exchange theory, was developed to analyze the factors associated with the number of online patients. A 3-wave data collection exercise, covering 4037 physicians on China’s Good Doctor website, was conducted during the months of February, April, and June 2017. Increases in consultation in a 60-day period were used as the dependent variable, whereas 2 series of data were analyzed using linear regression modeling. The fixed-effect model was used to analyze the 3-wave data.

**Results:**

The adjusted *R*^2^ value in the linear regression models were 0.28 and 0.27, whereas in the fixed-effect model, it was .30. Both the linear regression and fixed-effect models yielded a good fit. A positive effect of physicians’ effort on the aggregated number of online patients was identified in all models (*R*^2^=0.30 and *R*^2^=0.37 in 2 regression models; *R*^*2*^=0.23 in fixed effect model; *P*<.001). The proxies of physicians’ reputations indicated different results, with total number of page views of physicians’ homepages (*R*^2^=0.43 and *R*^2^=0.46; *R*^2^=0.16; *P*<.001) and number of votes received (*R*^2^=0.33 and *R*^2^=0.27; *R*^2^=0.43; *P*<.001) being seen as positive. Virtual gifts were not significant in all models, whereas *thank-you* messages were only significant in the fixed-effect model (*R*^2^=0.11; *P*=.02). The effort made by physicians online is positively associated with their aggregated number of patients consulted, whereas the effect of a physician’s reputation remains uncertain. The control effect of a physician’s title and hospital’s level was not significant in all linear regressions.

**Conclusions:**

Both the effort and reputation of physicians online contribute to the increased number of online patients’ consultation; however, the influence of a physician’s reputation varies. This may imply that physicians’ online effort and reputation are critical in attracting patients and that strategic manipulation of physician profiles is worthy of study. Practical insights are also discussed.

## Introduction

### Background

With the increasing popularity of Web 2.0 technologies, people are seeking health information more frequently online [1-6]. The internet has become a popular medium for obtaining medical treatment from physicians, for discussing and receiving medical advice, and for identifying symptoms experienced. It also offers many advantages for patients in comparison with the offline world, such as convenience, time saving, and reduced limitations on space and time. Since the early 2000s, consumers have been able to access the ratings of medical services and choose physicians through websites [2]. Physician-rating websites (PRWs) are an alternative and novel way for patients to obtain information about physicians before receiving consultation. PRWs collect and present information about patients’ experiences and whether they were satisfied with their encounters with physicians [7]. They provide patients with an opportunity to discuss their health conditions with physicians and rate his or her quality of service and the care provided [8,9]. The number of PRWs throughout the world is increasing [3], such as in the United States (RateMDs.com and Healthgrades.com), the United Kingdom (iWantGreatCare.org), and Germany (Jameda.de and AOKGesundheitsnavi.de). In China, haodf.com [5] and guahao.com [10] are rising in popularity.

The development of PRWs has made it easier and more flexible for patients to access information and consult with physicians before receiving medical services. From a patient’s perspective, most PRWs are perceived to be positive and can help improve the provision of services [11]. They can provide patients with information about physicians, especially in relation to service quality [11,12]. Globally, the number of people engaging and conversing on PRWs is increasing, with many using them to share their health care experiences, seek health care information, and rate the services received from health care practitioners [13,14]. The ratings on PRWs can also affect the patient care received from providers [15]. To make PRWs more reliable for the public, adjusting the content of PRWs to suit the differing information needs of health consumers is important [16].

People obtain information about the quality of physicians through the online view functionality built into PRWs. The information sought relates to the online efforts and reputation of physicians. Physician efforts imply the amount of time and energy that a physician spends online. A physician’s reputation is associated with a patient’s perception of the physician’s characteristics or qualities in general, for example, honesty, capability, and reliability, and it is usually demonstrated in the form of positive evaluation toward the health care service received by the patient. On the basis of the evaluation of these 2 metrics, patients become better informed and make more critical decisions when choosing their physician. An increasing amount of research has tested the effects of online effort and reputation on the sales of products and decision-making by consumers [17-20]. However, little analysis has been conducted into the factors that contribute to the selection of physicians on PRWs. To fill this gap, this paper undertakes an investigation into the choice of physicians by patients on PRWs and aims to explore the factors that affect a patient’s choice of a physician. The effects of physicians’ effort and reputation online are also tested. Data for this research were collected from the Good Doctor website in China, one of the biggest PRWs in the country. As searching for suitable new physicians or evaluating their current one is deemed important for patients, this study provides insights into understanding how patients make their decisions online. By using the results of this study, marketers and the designers of PRWs can better tailor their online services to the needs of patients. The findings also offer practical insights for online health care providers to encourage patients’ utilization of health care websites.

### Physician Efforts

The term *effort* was defined by Naylor et al [21] as “the amount of energy ‘spent’ on an act per unit of time.” In the sales and marketing domain, effort represents the amount of time and energy a salesperson devotes to the selling of a product or service, relative to another salesperson [22]. The effect of effort on performance has been considered by many researchers [22-24], with eﬀort being identified as a direct antecedent of performance in the research of salespeople [25]. As services are intangible, it is difficult to control their quality, compared with, for example, manufactured products. The verbal and nonverbal actions of employees have great influence on customers’ perceptions toward value and service quality [26]. The effort of an employee is more important in service settings as customer evaluations of service quality are often linked directly to the performance of the service provider. Social exchange theory (SET) is commonly used to explain the exchange behavior between various parties, with the aim of individual behaviors being to maximize satisfaction and minimize costs [27]. This also applies to the relationship of physicians and patients [28]. From the perspective of social exchange, the physicians’ participation in virtual communities is social exchange behavior, while patients can also provide social and economic returns for physicians as exchange returns [29].

An increase in visibility of effort from staff may generally lead to higher perceived quality by customers [26]. An employee’s effort, applied to their daily work, can influence consumer perception of the services being received [30]. If the employee is considered to pay extra effort to their work, then he or she may obtain a higher rating from their customers. The employee’s efforts will affect the research and purchasing intention of consumers, which is critical to a service organization’s overall performance [17]. This positive effect of effort may contribute to the consumer’s likelihood to browse and purchase goods in future. As online health care services are part of the service domain, a physician’s efforts, applied to the services they deliver, can affect a patient’s perception of quality of service and may change their choices and opinions toward physicians. The study by Liu et al [19] indicated that a physician’s effort was a positive indicator of online physician popularity, that is, physicians become more popular when they show a greater effort toward their service. When selecting physicians through PRWs, patients can visit the home page of the physicians and obtain additional information, such as their personal blog, published articles, and previous physician-patient communication. At this stage, patients gain a perception of the efforts made by the physician in the past, which may affect their attitude toward the physician and, thus, the likelihood of them selecting the physician. More effort shown by the physician online toward their service offerings may increase the chances of patients choosing them for consultation. Thus, we hypothesize the following:

H1: Patients prefer to consult with physicians that provide higher amounts of effort online.

### Physician Reputation

The definition of reputation varies in different fields of research. In the online marketplace, reputation is understood as a conditional probability that an individual will behave in a certain manner [31]. Early research on reputation focused generally on experimentation [32] as it was difficult to measure reputation in the offline world. With the development of the internet and online user-centered social tools, the ability to measure reputation has developed, with much research being conducted into the development of methods to measure reputation on e-commerce and eHealth websites. As the reputation of a person and/or website can help consumers and vendors make better decisions, communicate more effectively, and improve cooperation, it is now seen to play a major role in online service delivery [33]. Reputation is acknowledged as one of the most influential factors that affect a consumer’s behavior and seller’s performance in online marketplaces [34]. A high reputation contributes to reducing information asymmetry and the reduction of risk and uncertainty perceived by consumers [35]. In the domain of SET, reputation is taken as an important factor that affects the behavior of online patients [36]. Many researchers have explored the effects of online reputation on sales in fields such as tourism, the retailing of books, and online auctions. Previous research has demonstrated that a correlation exists between reputation and sales, generally with online reputation having a positive effect on sales. For example, Dewan and Hsu [37] found that reputation has a significant effect on the sales of products on customer-to-customer auction websites, using data available on eBay. Similarly, Ye et al [20] indicated that seller reputation has a positive impact on sales volume, following analysis of sales data from Taobao.com, a Chinese e-commerce website.

The online reputation mechanism serves as the basis for online transactions, and it helps consumers obtain more detailed information about products before purchase. As physicians know more about their service quality and patients’ health conditions than the patients themselves, information asymmetry can be considered severe in the online health care market [38]. Without PRWs, patients are unable to evaluate accurately the quality of a physician’s services before consultation; this may lead to a misunderstanding or misinterpretation of information relating to the physician. Similarly, online consultation experiences can reduce the risks caused by information asymmetry and build trust between the patient and physician [39]. As a result, online reputation mechanisms can also be applied in the delivery of medical services. For example, health services provided online can allow patients to share their experience quickly and objectively [40]. There are few studies relating to the reputation of online health care services. The study by Josang [40] indicated that sound reputation systems can be applied to medical services, whereas word-of-mouth from family and friends of patients is considered important when selecting an appropriate physician [41]. When selecting Web services, reputation plays an important role in a patient’s decision-making process [42], with reputation being treated as the most valuable attribute of a physician [43]. Reputation is also a vital quality factor in health care delivery as patients rely heavily on word-of-mouth when deciding which physician to approach [44]. The online reputation of physicians can help patients choose a suitable physician. If a physician is highly regarded, with a high reputation online, patients are more likely to consult with him or her; thus, we present the following hypothesis:

H2: Patients prefer to consult with physicians with a high level of online reputation.

## Methods

### Data Collection

Data were collected from the Good Doctor website (www.haodf.com) in China, one of the largest online PRWs in the country [5]. Currently, over 7500 hospitals and more than 500,000 physicians are active on the website. According to Good Doctor, online physicians are divided into 28 groups and more than 100 departments. As different medical departments provide varying treatment and patient-physician communication, we selected 1 department for our study to avoid interference from different departments. As China is facing the problem of an aging population [45], patients in the department of heart diseases represent a sizeable group; thus, we selected this department, which specializes in cardiovascular diseases. Using a Java-based program, we collected data from the homepages of 5996 cardiovascular physicians. Data were collected on February 25, April 27, and June 27, 2017, which allowed us to form a longitudinal panel dataset (there is a 60-day interval between these dates). We matched the URL and name information of physicians in 3 different stages. Following this process, it was revealed that 1361 physicians had data with less than 3 stages (among them, 698 physicians had 2 stages missing and 665 had 1 stage missing), 472 physicians had more than 1 null value across 3 stages, and 172 physicians had some abnormal values as the number of proxies decreased over time. The removal of samples with those missing values or abnormal values yielded a final dataset of 4037 patients from 878 specific hospitals. Approximately 46.54% (1879/4037) of the physicians were men, 22.94% (926/4037) were women, and 30.52% (1232/4037) could not be identified. The majority of physicians (3856/4037, 95.52%) are working in tertiary hospitals. Approximately 36.22% (1462/4037) of these physicians possess the title of Director. The details of the characteristics used in our study are listed in Table 1.

**Table 1:** Demographic characteristics of physicians. N=4037.

Physician characteristics		Statistics, n (%)
**Gender**		
	Male	1879 (46.54)
	Female	926 (22.94)
	Not reported	1232 (30.52)
**Hospital level**		
	Tertiary hospital	3856 (95.52)
	Secondary hospital	174 (4.31)
	One	7 (0.17)
**Professional title**		
	Director Physician	1462 (36.22)
	Associate Director Physician	1445 (35.79)
	Attending Physician	917 (22.71)
	Residing Physician	213 (5.28)

**Figure 1:**
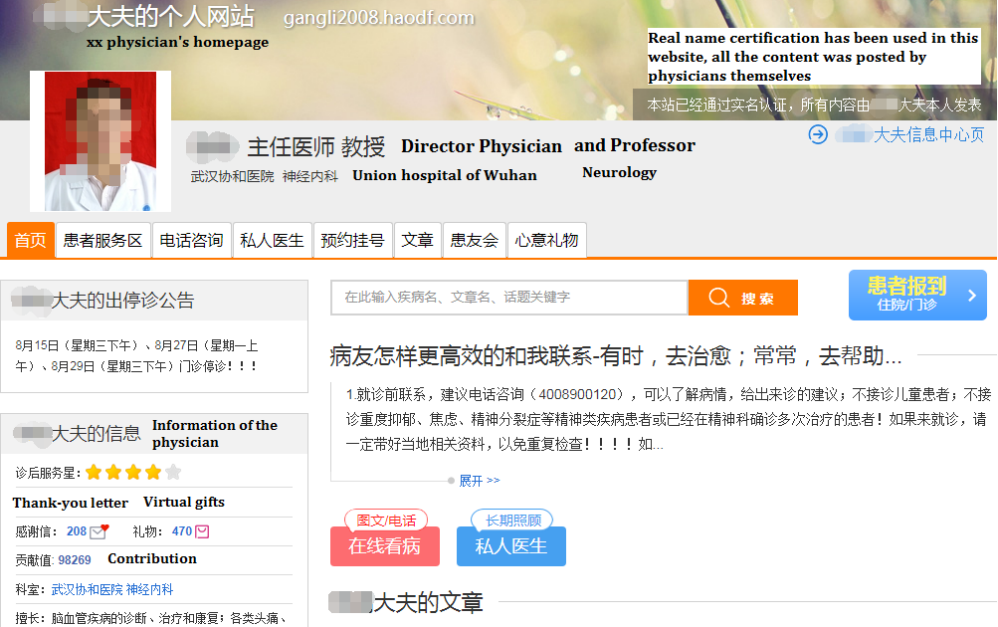
An example of a Physician’s Homepage (Accessed on August 29, 2018).

**Figure 2:**
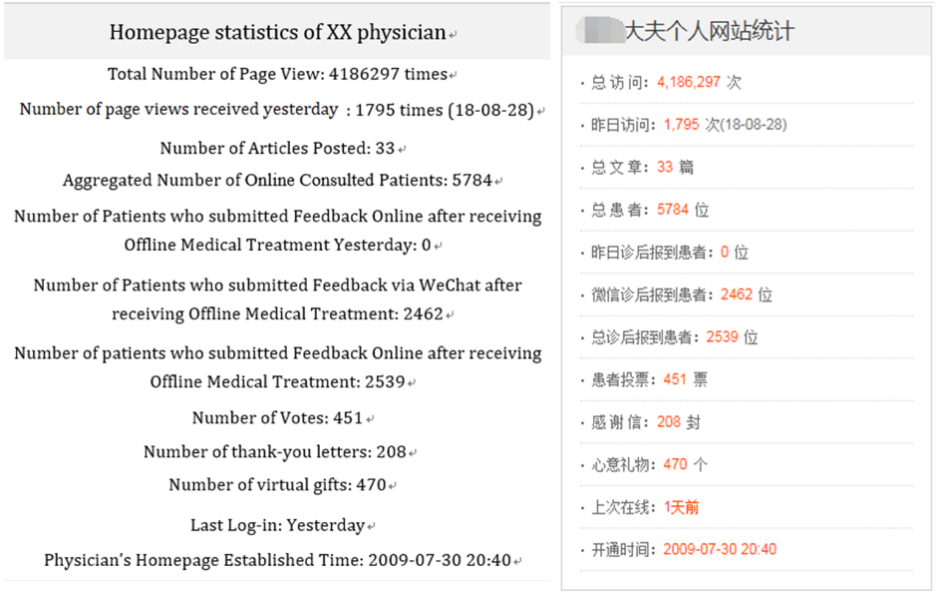
An example of the physician statistics Web page, calculated by the Good Doctor website (accessed on August 29, 2018).

The online homepages of physicians provide many types of information about physicians, including the total number of homepage views, number of votes, and number of thank-you messages and virtual gifts received. A screenshot from a physician’s homepage is presented in Figures 1 and 2, which show the different types of information available to the patient.

### Dependent Variable

The aggregated number of patients that had received online consultations in the 60-day interval can reflect the patient’s choice as interacting with a specific physician was a result of choice.

### Independent Variables

The online effort of a physician was estimated by their contribution to the Good Doctor website and patients; the website determines this value to express physician activity and engagement levels, which can help patients choose the most suitable physician. Physicians can affect the value of their contributions by updating their information in a timely manner, publishing articles to educate patients, and answering questions received from previously consulted patients. These behaviors can indicate how much effort the physician puts into online rating websites. As Campbell and Pritchard [46] indicated, as both the duration of time spent working and the intensity of work activities represent important aspects of effort, it is appropriate to choose this contribution score as its proxy.

**Figure 3:**
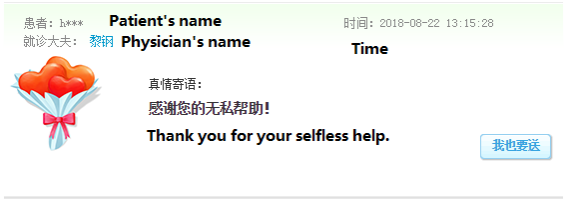
An example virtual gift.

**Figure 4:**
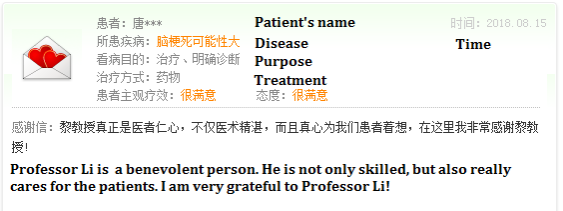
An example of the thank-you message.

The physician’s reputation possesses more measurement items than the previous. Generally, the persuasiveness of online consumer reviews depends on both their quality and quantity [47]. However, in the Good Doctor website, the star ratings only relate to the quality of service after diagnosis and treatment. The ratings are calculated by the website based on the number of patients diagnosed online and followed up online. A high proportion of patients followed up online will lead to more stars for the physician. If the physician diagnosed more than 100 patients online, he or she will get the star rating displayed on their homepage. As the star rating only refers to the followed-up element and only a small proportion of physicians gained this star rating, we have ignored the star rating in this study and only taken into consideration the quantity. We used the total number of page views shown on the physician’s homepage, number of votes, number of thank-you messages, and the number of virtual gifts received as proxies for physician reputation. The total number of homepage views reflects the physician’s fame, as online views are the first step and an indicator in learning about the physician. If patients are satisfied with the services provided by the physician, they can vote, write a thank-you message, or send virtual gifts to the physician. All options are free, except for virtual gifts; the website charges the patient for sending virtual gifts, ranging in price from several yuan to several hundred yuan. After deducting a small amount of website operating expenses, the fee will be allocated to the account opened by the physician for their time sacrificed. Table 2 provides sample data on physicians’ efforts, observed from the Good Doctor website. Figures 3 and 4 show examples of a virtual gift and thank-you message.

**Figure 5:**
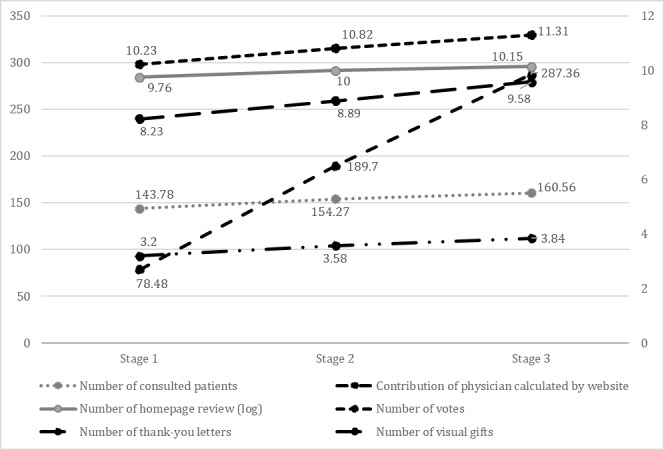
Statistics on the trend of different waves.

To present major changes of the variables in the 3 waves, the mean value was adopted to show their trend, as shown in Figure 5. For the number of contributions, 1400 was deducted from the value, to fit the size. A log transformation was taken for views as its numerical value was much larger than other values.

**Table 2:** Measurement items and statistics of variables.

Variable, proxy, and wave			Minimum number of variables	Maximum number of variables	Mean (SD)
**Number of consultations**					
	**Consultation number of patients in a 60-day period**				
		1	0	16,003	143.78 (546.82)
		2	0	17,620	154.27 (573.64)
		3	0	18,443	160.56 (589.00)
**Online effort of physician**					
	**Contribution of physician, calculated by the Good Doctor website**				
		1	0	210,580	1478.48 (6456.89)
		2	0	234,605	1589.70 (6837.36)
		3	0	251,440	1687.46 (7112.74)
**Online reputation of physician**					
	**Total number of homepage views (log)**				
		1	3.09	16.23	9.76 (2.00)
		2	5.78	16.27	10.00 (1.75)
		3	6.32	16.32	10.15 (1.66)
	**Votes**				
		1	0	1587	10.23 (38.83)
		2	0	1733	10.82 (41.38)
		3	0	1860	11.31 (43.52)
	**Thank-you messages**				
		1	0	680	3.20 (16.60)
		2	0	782	3.58 (18.36)
		3	0	863	3.84 (19.71)
	**Virtual gifts**				
		1	0	1956	8.23 (49.80)
		2	0	2137	8.89 (53.58)
		3	0	2424	9.58 (58.74)

### Control Variables

The control variables used in this study are (1) the physician’s title in the hospital and (2) the level of the hospital. The title of a physician indicates the position and responsibility of the physician in the hospital. It can also reflect the professional expertise of the physician. There are 4 titles used on the Good Doctor website: Director Physician, Associate Director Physician, Attending Physician, and Residing Physician. A higher title suggests greater responsibility on the part of the physician. Two dummy variables (0 or 1) were used to indicate Director Physician, Associate Director Physician, and Attending Physician, respectively. There is also a variable that can represent the standing of the hospital, that is, the ranking of the hospital in China. According to the *standard for the grade management of hospitals* in China, the ranking of hospitals can be divided into 3 levels: level 1 refers to hospitals that are basic and typically provide health care services to communities, level 2 refers to secondary hospitals, and level 3 are tertiary hospitals. Level 3 hospitals employ more staff and own a greater number of beds than the other 2 levels and are often considered to provide a higher quality of service. We also used 2 dummy variables to indicate hospital rankings, ranging from 3 to 2. In our research model, these variables were used to control the effects of a physician’s title and the ranking of hospitals on patient choices.

### Model Estimation

**Figure 6:**
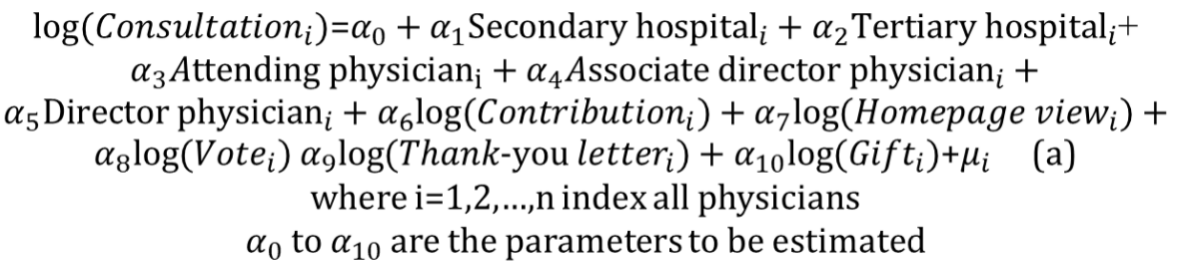
Formula of the regression model.

To test our hypotheses against the effects of a physician’s effort and reputation, we formulated a regression equation using the linear regression for each time series. We transformed the dependent variables and continuous independent variables into the log form, as the distribution of these variables may not be normal. The equation is separated into 2 stages, as shown in Figure 6(a).

Log(Consultation_i_) denotes the number of patients who consulted the physician in the 60-day period. Secondary hospital and tertiary hospital indicate hospital ranking, whereas the title of each physician is also included. Log(Contribution_i_) represents the effort of the physician online, whereas log(Hompage view_i_), log(Vote_i_), log(Thank-you message), and log(Gift_i_) indicate the number of homepage views, votes, thank-you messages, and virtual gifts received, respectively; these represent the online reputation of physicians. After log transformation, the response variable is approximately distributed (the result of P-P plots is approximate, with a straight diagonal line indicating the data are normally distributed [48]; more details are shown in Multimedia Appendix 1). μ_i_ is the error term. The physician’s title and the rank of the hospital are included as control variables.

Panel data analysis was also conducted, which is a widely used form of longitudinal analysis among social science researchers [49]. The panel data allowed for the control of unobservable variables that change over time and permitted the study of dynamics of change with time series. Meanwhile, the panel data controlled the variables that could not be observed or measured in each group (eg, gender) and the unobservable variables that changed over time but not across entities (eg, physicians with different titles). The panel data allowed for the inclusion of the variables at different levels of analysis.

**Figure 7:**
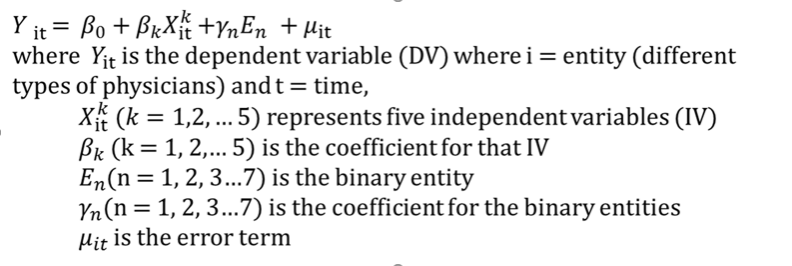
Formula of the fixed-effect model.

In this study, gender, the physician’s title, and the hospital level were chosen as the level of analysis. The following fixed-effect model was set up to explore the relationship between effort and reputation factors and physician’s consultation within each type of physician, as something within the physician groups may impact or bias the predictor or outcome variables; this needed to be controlled in the model. The key insight is that if the unobserved variables do not change over time, then any changes in the outcome variable must be caused by influences other than the fixed characteristics. As such, once the effect of the time-invariant characteristics from the predictor variables is removed, we can assess the predictors’ net effect on outcome variables. Generally, 2 approaches to build the fixed-effect model are highlighted, with the binary variable option being chosen as it allows for the separation of the association of the number of consultations and other individual factors. As 3 groups of entities were used (gender, physician’s title, and hospital level) to generate binary (dummy) variables, 7 entities from 3 groups were presented in the final model, accordingly (see Figure 7).

## Results

### Results of the Linear Regression

SPSS 19.0 (IBM) and Stata 12.0 (IBM) were used to analyze the data collected. Table 3 presents the results by ordinary least squares. Equations are presented in hierarchical order. First, the results are shown with only control variables in columns 1a and 1b. Then, the independent variables are added to columns 2a and 2b. The adjusted *R*-square and *F* value both indicate a good fit. The results of the VIF (Variance Inflation Factor) statistics for the variable indicate no multicollinearity (the VIF statistic of every variable is not greater than 10). Not all factors indicated a significant effect. The coefficient of contribution (B1=0.30, B2=0.37; *P*<.001), total number of homepage views (B1=0.43, B2=0.46; *P*<.001), and votes (B1=0.33, B2=0.27; *P*<.001) were all positive and significant. However, as thank-you messages (*P*=.07) and virtual gifts (*P*=.23) were not significant, the hypothesis could not be confirmed.

### Results of the Fixed-Effect Model

Table 2 and Figure 5 show a numerical growth of each independent variable. To take the physician’s individual factors into consideration, the fixed-effect model of panel data analysis was applied, as shown in Table 4. The effect of thank-you messages is seen to be significant, after the control of individual factors. Hypothesis 1 predicted that patients prefer to choose online physicians with greater effort. The results in Tables 3 and 4 support this hypothesis, as the coefficient of contribution (B1=0.30, B2=0.37; B3=0.19; *P*<.001) was positive and significant. Hypothesis 2 indicated a significant influencing path between the online reputation of the physician and patients’ choice on physicians. Hypothesis 2 can be considered partly supported as the coefficient of number of homepage views (B1=0.43, B2=0.46; B3=0.18; *P*<.001) and number of votes (B1=0.33, B2=0.27; B3=0.48; *P*<.001) are significant, whereas thank-you messages are only significant in the fixed-effect model (B3=0.17; *P*=.01). However, virtual gifts (*P*=.11) were not significant in all models. The relationship between the online reputation of the physician and the patient’s choice of physician is uncertain and needs further analysis. All control variables are not significant in the linear regression models. In the fixed-effect model, the physician’s title effect is confirmed as physicians with the title of Associate Director Physician and Director Physician are more likely to be consulted with than the physician with the title of Residing Physician.

We further predicted a significant relationship between the online effort of physicians’ and patients’ choices of physicians. Tables 3 and 4 provide support for this hypothesis as the coefficient of contribution in all 3 models was positive and significant. Therefore, we can posit that the online effort of physicians has a positive impact on a patient’s choice of physician. We assume that patients would like to consult with a physician who has a good online reputation in hypothesis 2. However, this assumption is only partly supported because of thank-you messages and virtual gifts being not significant.

**Table 3:** Linear regression results of model 1.

Variables		Number of consultations during the 60-day period^a^, coefficient (95% CI)			
		Model 1a	Model 1b	Model 2a	Model 2b
**Control variables**					
	Attending Physician	0.13 (−1.143 to 1.406)	0.41 (−0.689 to 1.504)	-1.94 (−4.941 to 1.068)	−0.79 (−3.386 to 1.809)
	Associate Director Physician	0.82 (−0.430 to 2.085)	0.74 (−0.346 to 1.820)	−1.67 (−4.666 to 1.327)	−0.76 (−3.345 to 1.831)
	Director Physician	0.81 (−0.076 to 1.701)	0.78 (−0.303 to 1.859)	−1.37 (−4.364 to 1.624)	−0.66 (−3.247 to 1.923)
	Secondary hospital	0.76 (−0.313 to 1.830)	0.60 (−0.165 to 1.369)	−0.90 (−4.017 to 2.215)	−0.21 (−2.909 to 2.484)
	Tertiary hospital	1.07 (−0.181 to 2.325)	0.70 (−0.069 to 1.459)	0.17 (−2.824 to 3.164)	0.35 (−2.238 to 2.947)
**Independent variables**					
	Contribution (log)	—^b^	0.30^c^ (0.214 to 0.375)	—	0.37^c^ (0.254 to 0.476)
	Total number of homepage views (log)	—	0.43^c^ (0.335 to 0.536)	—	0.46^c^ (0.358 to 0.564)
	Number of votes (log)	—	0.33^c^ (0.206 to 0.447)	—	0.27^c^ (0.134 to 0.398)
	Thank-you messages (log)	—	0.07 (−0.114 to 0.258)	—	0.17 (−0.015 to 0.354)
	Virtual gifts (log)	—	0.12 (−0.082 to 0.326)	—	0.14 (−0.073 to 0.347)
Constant		0.84 (−0.597 to 2.282)	2.90^c^ (1.462 to 4.345)	3.57 (−0.664 to 7.799)	3.88^d^ (0.163 to 7.591)

^a^Model 1a: *R*^2^=0.05, goodness of fit (F_4037_)=9.27. Model 1b: *R*^2^=0.28 and goodness of fit (F_4037_)=33.38. Model 2a: *R*^2^=0.03 and goodness of fit (F_4037_)=4.12. Model 2b: *R*^2^=0.27 and goodness of fit (F_4037_)=27.64.

^b^Not included in the model.

^c^*P*<.01.

^d^*P*<.05.

**Table 4:** Results of fixed-effect model test (model 2).

Variables^a^	Number of patients consulted during the 60-day period (log)		
	Coefficient	*t* value (degrees of freedom)	*P* value
Contribution (log)	0.19	16.21 (4037)	<.001
Total number of homepage views (log)	0.18	8.04 (4037)	<.001
Number of votes (log)	0.48	40.36 (4037)	<.001
Thank-you messages (log)	0.17	11.82 (4037)	.01
Virtual gifts (log)	−0.06	1.13 (4037)	.17
Male	−0.02	−0.65 (4037)	.52
Female	0.01	0.31 (4037)	.76
Attending Physician	0.17	1.79 (4037)	.07
Associate Director Physician	0.22	2.33 (4037)	.02
Director Physician	0.30	3.15 (4037)	<.001
Secondary hospital	−0.07	−0.22 (4037)	.82
Tertiary hospital	−0.12	−0.37 (4037)	.71
Constant	−1.32	−3.82 (4037)	<.001

^a^Please note that the value of *F* test that all μ_it_ =0 (3643) is 149.15 (*P*<.001) and *R*^2^=0.82.

## Discussion

### Principal Findings

The main purpose of this study was to examine the relationship between the online effort of physicians (contribution), physician reputation (total number of home page views, number of votes, number of thank-you messages, and virtual gifts received), and patients’ choices of physicians on the health care website, Good Doctor. Similar to previous research [19,34,50], the predictions are tested through the data collected from the online health website. However, we then introduced physician effort and reputation and observed the data in 3 waves. To ensure the credibility of the stated results, we conducted 2 analyses and presented their differences in the results.

First, our results indicate that patients are more likely to consult with physicians online who show greater effort. The online effort of a physician was represented by the figure of reputation online, which can be increased by the physicians themselves by revising their personal information regularly, publishing educational articles for patients on their homepage, and answering questions posed by previously consulted patients. These actions can present a positive and hard-working image of a physician on their website. Patients would take these factors into consideration when seeking medical consultation. Liu [19] also suggested that physician efforts would influence patients. From the perspective of SET, researchers have focused on the consumers’ effort paid on learning how to use or using online services [51], whereas little attention has been paid to the service providers. We confirm that the physician’s effort is also important when providing services. The website’s marketers and the physicians themselves should pay more attention to information relating to their effort if they wish to attract more patients for consultation. For example, health care websites can develop an *effort mechanism* whereby physicians can strive to achieve higher scores that are then displayed on their personal homepage.

Second, the effect of physicians’ online reputations on the patient’s choice is complex. Reputation is taken as an important part from SET. When researchers explore the behavior of online users and patients [36,52], it is a critical benefit for the online users. However, results indicate that not all reputational factors generate similar effects. Some of the factors, such as total number of homepage views and number of votes received, represent a significant result, and the coefficient indicates a positive influence. However, the effect of thank-you messages and virtual gifts is not always significant. These results are not consistent with the study by Yang et al [50]. These differences may be caused by the variances in sample size and time selection, as the study by Yang et al [50] was conducted in 2013, had a comparatively smaller sample size, and was not limited to a specific department. It may also suggest that the physicians in our study have already established their personal reputation through online efforts. As time proceeds, their reputation is less likely to rely on the result of thank-you messages and virtual gifts. In addition, cardiovascular disease patients can be difficult to please because of the nature of their chronic disease, which may also contribute to the insignificance. The coefficient of virtual gifts is not significant as patients must log in to the Good Doctor website and pay additional money for these virtual gifts, and consequently, the gift may not represent the true reputation. As we already understand that reputation is important for offline physicians [53], we are now convinced that it also makes sense in the online world. There are many factors that can reflect reputation; identifying the differences and the characteristics of these factors should be considered critical.

Third, the control effects of the level of hospital and title of physician are not seen as significant in the linear regression model. Level 1 hospitals (0.17%, 7/4037) are much less significant than those categorized as secondary hospitals or tertiary hospitals. Physicians with the title of Residing Physician are also in a lower proportion (5.28%, 213/4037), which might have led to their result being not significant. However, the result from the fixed-effect model indicates a significant effect for physician’s title. The different result with the regression model may be attributed to bias of data from only 1 period. Patients prefer physicians with the title of Associate Director Physician and Director Physician to the title of Residing Physician. The online platform may weaken the impact of gender and hospital and allow patients to focus more on the ability of the physicians.

Finally, the results also suggest a recurring problem, which was identified in the information asymmetry domain. Arrow [54] first proposed the concept of *information inequality* in the research of physicians and patients in medical markets, stating that an information problem exists in the relationship between patients and physicians. Patients try to obtain and analyze information before choosing a suitable physician. As a service provider, this common goal of information requirements by patients should be satisfied and lessen the divide between health supply and demand. Health care providers can offer more channels for both patients and physicians to express themselves, such as tailored functions to make inquiries, release detailed information for physicians, explain and quash possible misunderstandings, and try to maintain a positive image of the physician.

### Conclusions

This paper explores the effects of physicians’ online efforts and their reputation on patients’ decision-making when choosing to consult with a physician. We used linear regression and fixed-effect modeling to test our hypotheses. Some of our assumptions have been proven by the results identified; the physicians’ effort is positively associated with patients’ decision-making, whereas the effect of a physician’s reputation remains uncertain, with 1 or 2 negative items being identified. The differences in the results of linear regression and fixed-effect models indicate the importance of time factor and method selection.

The findings assist us in understanding the effects of such information on a patient’s choice of a specific physician and, thus, contribute to the field of online health care research. By exploring the relationship between online physicians’ factors and patients’ choices of physicians, we contribute to the scarce literature relating to the online choice of physicians. It should be noted that the online information of physicians can affect the choice of patients. This research can help online health care providers and marketers to make strategic decisions on what information to display online to attract and retain a greater number of patients. The findings also extend the research of SET in the field of online health. More attention should be paid to the behavior of physicians.

Our findings also identify some limitations. First, data were collected solely from the Good Doctor website in China, and therefore, the findings may not be generalizable to other health care websites in other countries. Future research could collect and analyze data from multiple websites from different countries. Second, research must be conducted over a much longer period. The data used in this study were collected in 3 phases within a 4-month period only; we will continue the collection of data as part of our further study. Third, although the website confirms that the information about physicians was provided by the physicians themselves, we cannot guarantee that was always the case in reality. Finally, the variables of physician reputation and effort can be illustrated and measured by other items; for example, Wu and Lu [10] indicated that the reputation of one physician’s colleagues would affect the quantity of a focal physician’s future view.

## Appendix

Multimedia Appendix 1 P-P plot of variables.

## Abbreviations

PRWs: physician-rating websites
SET: Social Exchange Theory
