# Supplementary figures and images for: The Effect of Online Effort and Reputation of Physicians on Patients’ Choice: 3-Wave Data Analysis of China’s Good Doctor Website
Source: J Med Internet Res. 2019 Mar 8;21(3):e10170. doi: 10.2196/10170 (PMC6429049; doi:10.2196/10170)

## Appendix

P-P plot of variables

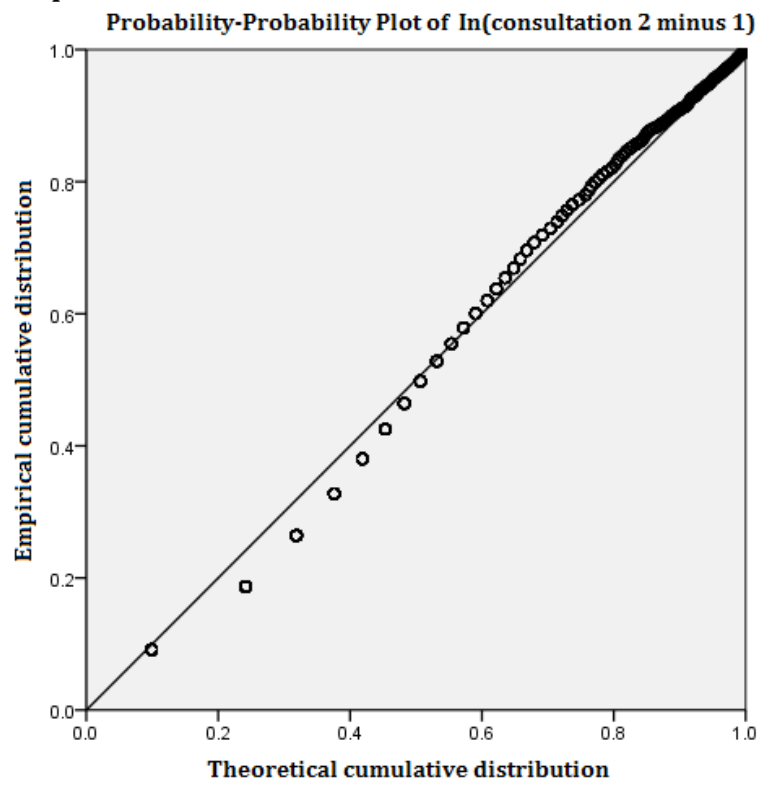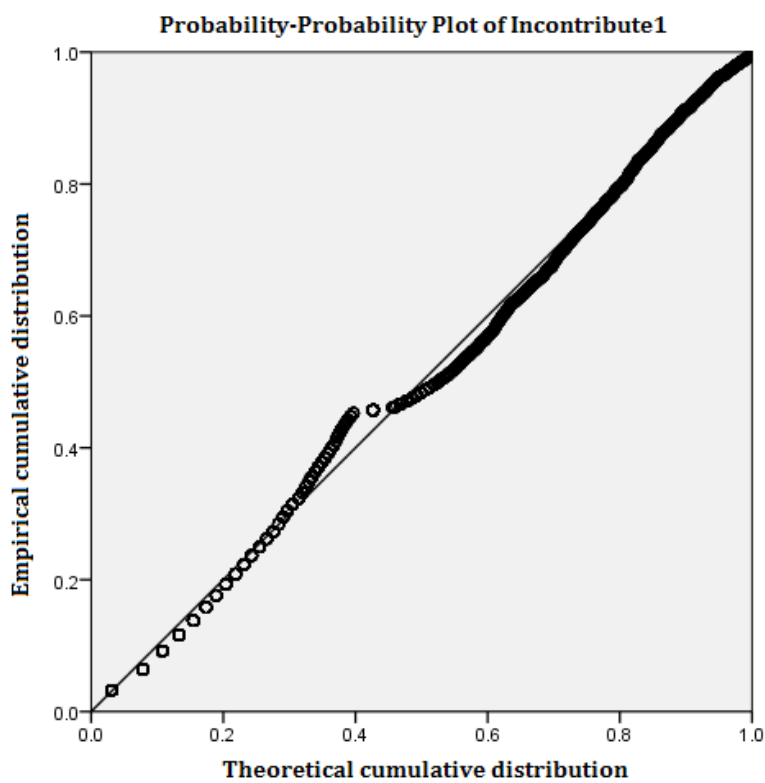

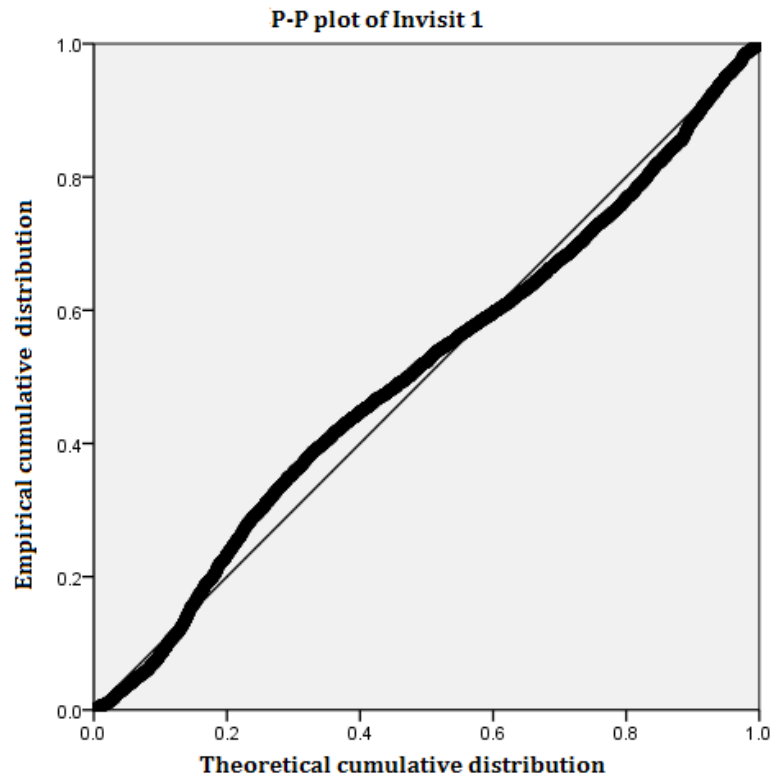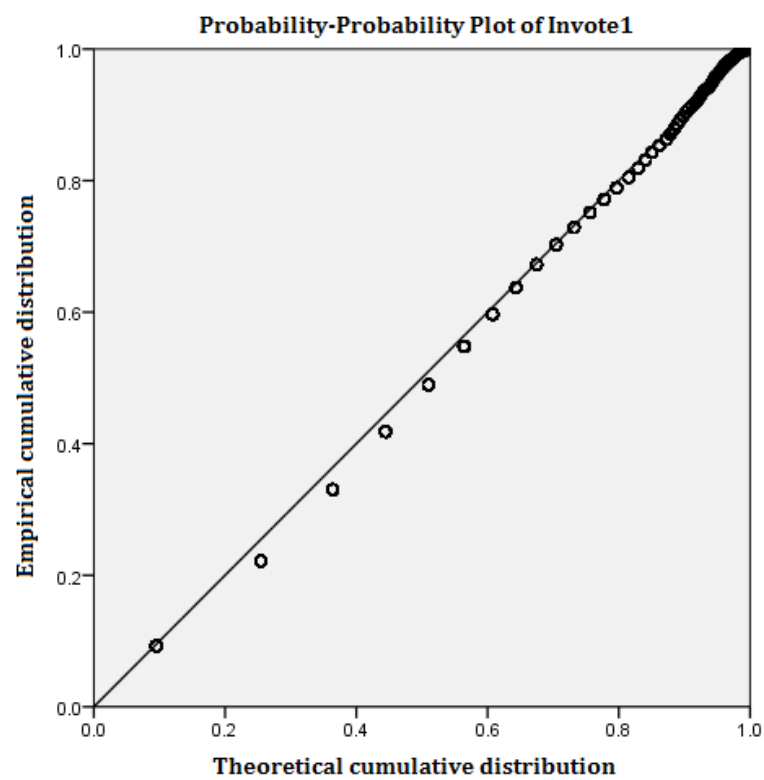

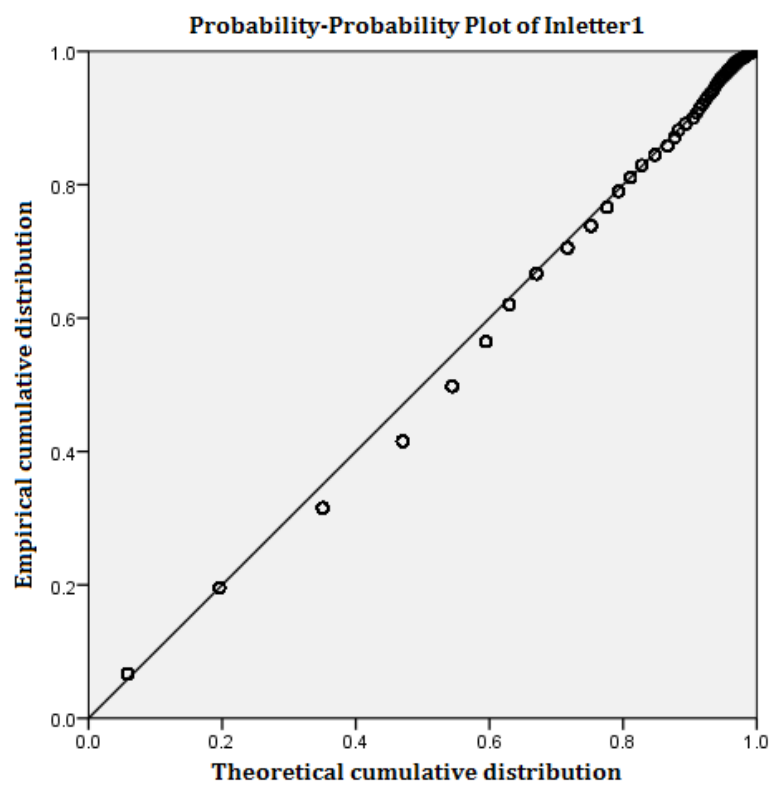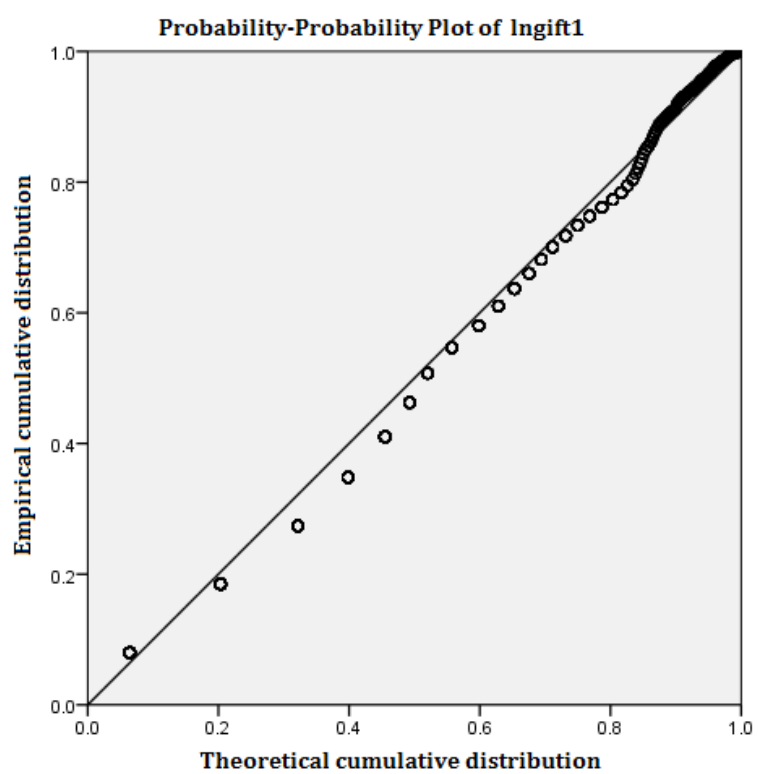

Supplement: Multimedia Appendix 1 [file jmir_v21i3e10170_app1.pdf]
